# Social capital and the number of children’s cafeterias (*kodomo shokudo*) in Japan: a longitudinal ecological analysis

**DOI:** 10.1265/ehpm.25-00366

**Published:** 2026-07-15

**Authors:** Hiroshi Murayama, Takumi Suda, Takahiro Tabuchi

**Affiliations:** 1 Research Team for Social Participation and Healthy Aging, Tokyo Metropolitan Institute for Geriatrics and Gerontology, 35-2 Sakae-cho, Itabashi-ku, Tokyo 173-0015, Japan; 2 Graduate School of Medicine, Tohoku University, 2-1 Seiryo-cho, Aoba-ku, Sendai, Miyagi 980-8575, Japan; 3 Cancer Control Center, Osaka International Cancer Institute, 3-1-69 Otemae, Chuo-ku, Osaka 541-8567, Japan

**Keywords:** Children’s cafeteria, Kodomo shokudo, Social capital, Ecological study, Longitudinal design, Japan

## Abstract

**Background:**

In Japan, community-driven dining spaces known as children’s cafeterias (kodomo shokudo) have increased rapidly. Despite their rapid expansion, the role of social environmental factors in the development of children’s cafeterias, particularly social capital, has received limited empirical attention. We examined the association between social capital and changes in the number of children’s cafeterias in Japan.

**Methods:**

We conducted a longitudinal ecological study. The outcome was the change in the number of children’s cafeterias per municipality between 2020 and 2022. Social capital indicators (trust in neighbors, norm of reciprocity, trust in the national government, neighborhood ties, and social participation) were derived from a nationwide internet survey.

**Results:**

A total of 512 local municipalities were analyzed. Multiple regression analyses showed that higher neighborhood trust was positively associated with increases in the number of children’s cafeterias, whereas denser neighborhood ties were inversely associated with such increases.

**Conclusions:**

The findings suggest that cognitive and structural dimensions of social capital may operate differently in the diffusion of children’s cafeterias. These results indicate that community social contexts are associated with the spread of child-focused initiatives.

**Supplementary information:**

The online version contains supplementary material available at https://doi.org/10.1265/ehpm.25-00366.

## Introduction

In Japan, children’s cafeterias (*kodomo shokudo* in Japanese)—community-driven dining spaces for children—have garnered increasing attention. A nationwide survey which was conducted by a certified nonprofit organization, Japan Kodomo Shokudo Support Center Musubie (hereafter, Musubie) reported that public awareness of children’s cafeterias is 87.7% among the general Japanese population aged 15–79 years in 2023 [1]. These establishments offer children complimentary or affordable meals, addressing issues such as children dining alone owing to the extended work hours of parents and the financial challenges families face in providing proper meals, contributing to their expansion. Originating in the 2010s, children’s cafeterias have increased throughout the nation, with their numbers expected to exceed 9,000 by 2023, an increase of approximately 1,800 from 2022 [2], and an approximate 29-fold rise since 2016. Typically, these cafeterias operate at least once a month, run by volunteer and non-profit organizations, and are supported by both the Japanese and local governments, reflecting a communal commitment to the welfare of children.

Children’s cafeterias aim to offer nutritious meals at no cost or for a nominal fee to economically disadvantaged children, prevent children from eating alone, and provide nutritional education to bridge the nutritional gap among youngsters. Eating with others positively affects children’s dietary habits and body weight control [3, 4]. Furthermore, numerous cafeterias welcome not only children but also their parents and other community members, fostering intergenerational interaction and strengthening local social ties. In practice, many children’s cafeterias function as community hubs where residents gather, volunteer, and build social connections beyond meal provision. A qualitative study with cafeteria staff reported that children’s cafeterias influence not only children’s nutrition and dietary habits but also those of parents and other community members, highlighting their broader role as community-based social resources [5]. Accordingly, children’s cafeterias function not only as child-targeted services but as community-level initiatives embedded within the broader social environment. In addition, earlier research has also focused on people’s recognition of children’s cafeterias. For example, a study investigated factors related to the utilization of children’s cafeterias among parents of elementary school/junior high school students and found that lower-income household members were more willing to use the cafeterias [6].

However, the understanding of social environmental factors that shape the development of children’s cafeterias remains under-researched, with social capital being a potential factor. Social capital as defined by Kawachi and Berkman [7], refers to the resources available to individuals because of their membership in a network or group. Evidence suggests that higher social capital within a community can enhance the effectiveness of public services and the availability of local facilities and resources [8, 9]. Regarding children’s cafeteria, a previous cross-sectional study reported that higher social capital is related to a greater number of children’s cafeterias using a cross-sectional ecological design at the prefecture level [10]. This study suggests that social capital in the community may encourage the growth of children’s cafeterias. However, there are limitations. First, the analysis focuses on all 47 prefectures in Japan. An unresolved debate exists regarding the appropriate analytic unit for social capital. However, as the number of children’s cafeterias largely varies based on local municipalities, a more appropriate analytic unit of social capital may be a local municipality rather than a prefecture. Second, cross-sectional designs capture only a single moment in time, limiting their ability to elucidate causal relationships. Longitudinal designs are crucial for gaining a deeper understanding of these relationships.

To overcome these limitations, this study employed a longitudinal design to explore the association between social capital and the number of children’s cafeterias in Japan. We hypothesized that communities with higher social capital are more likely to see the development of children’s cafeterias, based on previous research [10]. Although the number of children’s cafeterias has increased substantially, their distribution remains uneven across municipalities. National policy frameworks in Japan (e.g., those of the Ministry of Health, Labour and Welfare and the Children and Families Agency), as well as Musubie, continue to emphasize further dissemination and sustainability of these community-based initiatives. Thus, understanding the social conditions associated with their spread remains an important research question.

## Methods

### Study design

This study used a longitudinal ecological design at the local municipality level using separate data sources for outcome and exposure variables. The outcome variable, the number of children’s cafeterias, was based on a national survey conducted by Musubie, in 2020 and 2022. We obtained data from Musubie, which included the number of children’s cafeterias in all local municipalities in Japan (n = 1,718, as of April 2020).

The exposure variable, social capital, was sourced from the Japan “COVID-19 and Society” Internet Survey (JACSIS), a nationally representative survey conducted online via a self-administered questionnaire. This survey was carried out from August 25 to September 30, 2020, using the services of a large internet survey agency (Rakuten Insight, Inc., Tokyo, Japan). Initially, it included 28,000 individuals between the ages of 15 and 79. After excluding 2,518 participants owing to inconsistent or suspected fraudulent responses, the sample size was adjusted to 25,482 valid respondents. The JACSIS study protocol was approved by the Research Ethics Committee of Osaka International Cancer Institute on June 19, 2020 (approval number: 20084). Informed consent was obtained from all subjects involved in the study. The present secondary analysis was also reviewed and approved by the Ethics Committee of the Tokyo Metropolitan Institute for Geriatrics and Gerontology on November 11, 2024 (approval number: R24-074).

The respondents of the JACSIS study resided in 1,261 local municipalities. However, 749 of these municipalities had fewer than 10 respondents. As social capital was measured by aggregating individual responses within municipalities, a small number of respondents results in a large variation in values. Therefore, we excluded the respondents from these 749 municipalities and included only those from the remaining 512 municipalities.

### Measures

#### Number of children’s cafeterias

We calculated the number of children’s cafeterias per 1000 individuals in each local municipality for both 2020 and 2022. In the analysis, we used the difference between these two numbers (“the number in 2022” minus “the number in 2020”) as the outcome variable.

#### Social capital

Following the methodology of the previous study [10], we generated five indicators of social capital and used them as the exposure variables in our analysis. Three indicators pertained to the cognitive dimension, including trust in neighbors, norm of reciprocity in the neighborhood, and trust in the national government. The remaining two indicators were derived from the structural dimension of social capital and included neighborhood ties and social participation.

In the JACSIS survey, cognitive social capital was assessed using single-item questions for each indicator: “people in your neighborhood can be trusted” (trust in neighbors), “people in your neighborhood help each other” (norm of reciprocity), and “national government can be trusted” (trust in the national government). Responses were recorded on a four-point Likert scale (1 = “agree,” 2 = “somewhat agree,” 3 = “somewhat disagree,” or 4 = “disagree”). For structural social capital, neighborhood ties were measured by the frequency of connections with neighbors on a seven-point scale: 1 = “never,” 2 = “once a month,” 3 = “2–3 times in a month,” 4 = “once a week,” 5 = “2–3 times in a week,” 6 = “4–5 times in a week,” or 7 = “almost every day (6–7 times in a week).” Responses indicating once or more frequent contact (i.e., responses of 4–7) were categorized as dense neighborhood ties. Finally, social participation was assessed by asking respondents whether they participated in activities such as volunteering, sports, and hobbies.

To generate the social capital indicators at the local municipality level, we aggregated individual responses for each of the five social capital items within the residential municipalities of the respondents. We calculated the proportions of people who agreed (i.e., responses of 1 and 2) to each cognitive social capital item, those who had dense neighborhood ties, and those who participated in at least one group activity by local municipalities. There might be a difference between the respondents of the internet survey and the representative Japanese sample. To account for potential selection bias, we applied inverse probability weights derived from logistic regression models that accounted for sex, age, and socioeconomic factors. These weights were constructed to reduce discrepancies between participants in the web-based survey and the population-based sample from the 2016 Comprehensive Survey of Living Conditions [11], which is representative of the Japanese population. These weights were applied at the individual level prior to aggregation, and municipality-level social capital indicators were calculated using weighted proportions. In the analysis, we divided the weighted municipality-level social capital indicators into tertiles: T1 (lowest), T2 (middle), and T3 (highest).

#### Community characteristics

Community characteristics of each local municipality were conceptually grouped into four domains: i) demographic structure, ii) area-level socioeconomic deprivation, iii) local labor force structure, and iv) institutional support capacity. All variables of community characteristics were measured as of 2020.

Demographic structure included population density (calculated by dividing the population size by the area of habitable land [12]) and the proportion of young residents (aged 0–14 years) [13]. Area-level socioeconomic deprivation was assessed using indicators of educational attainment (proportion of individuals who graduated from university or graduate school [13]), household vulnerability (proportion of single-parent households and single-person households [12]), and local economic disadvantage (unemployment rate [12]). Local labor force structure was captured by indicators of employment composition, including the proportion of workers engaged in the tertiary industry [12] and the proportion of workers employed within their residential municipality (calculated as the number of workers employed within the municipality divided by the total number of workers residing in the municipality [12]). Institutional support capacity was proxied by municipal-level social welfare expenditure [12] to reflect local policy capacity and institutional support that may influence the establishment and sustainability of children’s cafeterias.

### Statistical analyses

Multiple regression analysis was performed with the difference in the number of children’s cafeterias per 1000 individuals between 2020 and 2022 as the outcome variable. The modeling strategy included two models: Model 1, where the five social capital variables were separately added, and Model 2, where all social capital variables were added together (i.e., mutually adjusted). All indicators for community characteristics were controlled in both models. In addition, the number of children’s cafeterias in 2020 was adjusted in the model, with this variable log-transformed owing to its skewed distribution in the regression analysis. Results were presented as non-standardized regression coefficients (b), their 95% confidence intervals (CIs), and standardized regression coefficients (β). The analysis was performed using IBM SPSS 29 (IBM Corp., Armonk, NY, USA).

## Results

**Fig. 1:**
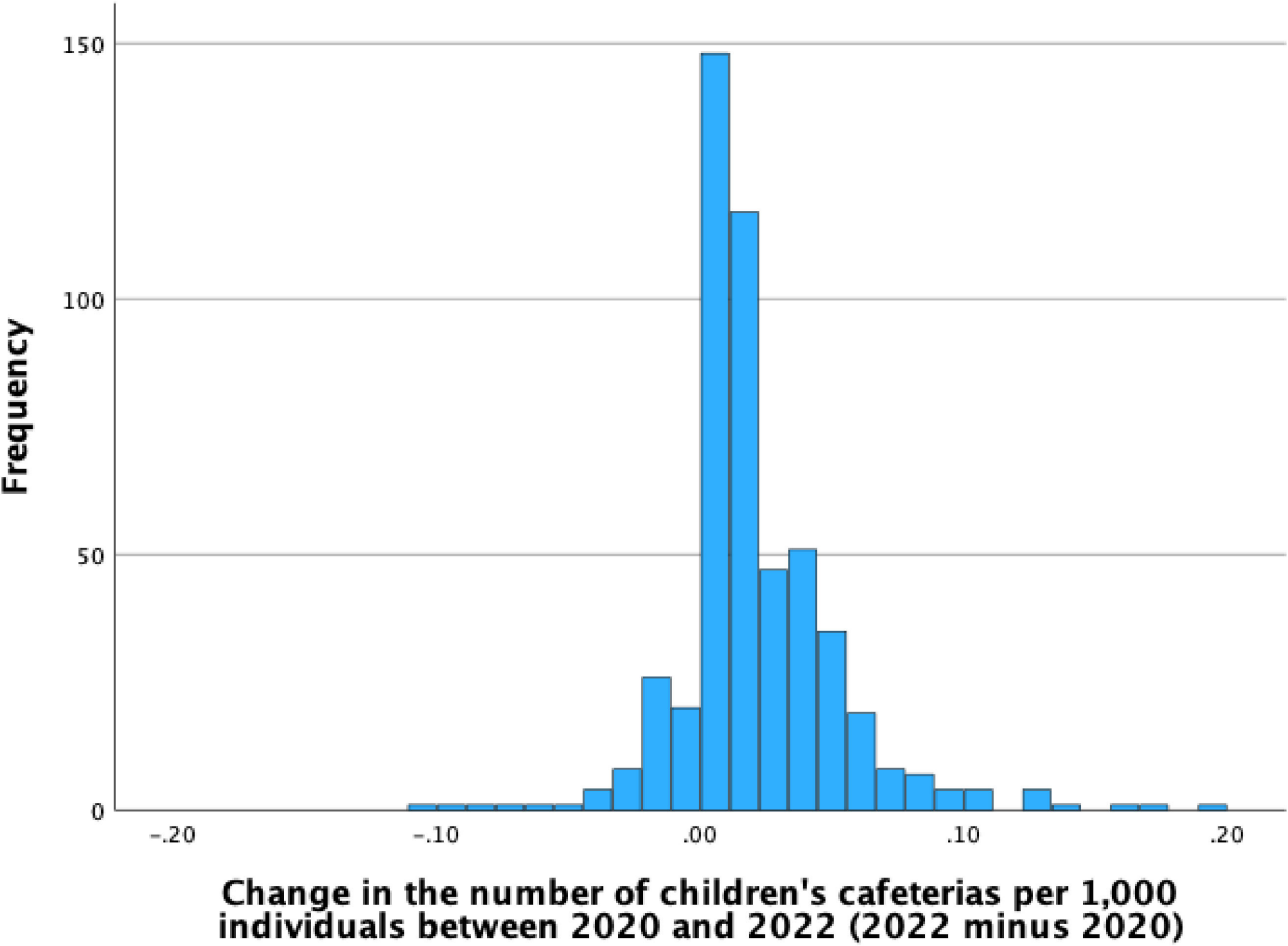
Histogram of the number of children’s cafeterias per 1,000 individuals between 2020 and 2022.

Table 1 shows the descriptive statistics of the variables in the analyzed local municipalities (n = 512). Figure 1 shows the distribution, which was approximately normal (skewness = 1.1; kurtosis = 5.2). Although the average increase in the number of children’s cafeterias per 1000 individuals between 2020 and 2022 was modest (0.02 per 1,000 population), this represented approximately a 50% increase relative to the 2020 baseline. Substantial variability was observed across municipalities (range: −0.10 to 0.19), indicating uneven diffusion of children’s cafeterias. Social capital indicators also showed wide variability across municipalities, suggesting considerable contextual differences in community environments.

**Table 1:** Descriptive statistics of variables.

	**Mean ± SD**	**Range** **(Min, Max)**	**Median**
Number of children’s cafeterias per 1000 individuals in 2020	0.04 ± 0.04	(0.00, 0.25)	0.03
Number of children’s cafeterias per 1000 individuals in 2022	0.06 ± 0.05	(0.00, 0.43)	0.05
Difference in the number of children’s cafeterias per 1000 individuals between 2020 and 2022	0.02 ± 0.03	(−0.10, 0.19)	0.02
Trust in neighbors (%)	7.0 ± 11.2	(0, 98.5)	4.3
Norm of reciprocity in the neighborhood (%)	5.4 ± 10.9	(0, 98.5)	1.9
Trust in the national government (%)	4.1 ± 8.5	(0, 83.5)	0.0
Neighborhood ties (%)	11.1 ± 13.4	(0, 94.7)	7.7
Social participation (%)	21.7 ± 17.4	(0, 98.5)	18.7
Population density of habitable land (individuals/km^2^)	2966.6 ± 4094.0	(117.5, 23182.1)	1246.8
% people aged 0–14 years	12.1 ± 1.7	(8.2, 20.7)	12.0
% people who graduated from university or graduate school	18.3 ± 6.5	(5.3, 43.2)	17.4
% single-parent households	9.4 ± 1.3	(0.5, 1.4)	9.5
% single-person households	33.6 ± 7.4	(18.0, 68.0)	32.7
Unemployment rate (%)	3.8 ± 0.7	(2.4, 6.0)	3.7
% workers in the tertiary industry	67.7 ± 8.9	(44.7, 87.2)	67.6
% workers employed within their municipality of residence	57.6 ± 18.9	(22.0, 99.0)	55.4
Social welfare expenditure (million yen)	8132.3 ± 14524.4	(257.0, 172775.6)	4091.9

SD: standard deviation.

We compared the characteristics of included and excluded municipalities. Although minor differences were observed, the overall distributions of key variables were generally similar (Supplementary Table 1).

Table 2 presents the association between social capital and the change in the number of children’s cafeterias. The characteristics of municipalities across tertiles of social capital indicators are presented in Supplementary Tables 2–6. A high level of neighborhood trust (T3) was significantly associated with an increase in the number of cafeterias compared to the lowest (T1) in Model 1 (b [95% CI] = 0.008 [0.000, 0.016]). The association remained similar in magnitude and direction after mutual adjustment in Model 2 (0.009 [−0.001, 0.019]; p = 0.072), suggesting a positive tendency. Conversely, a high density of neighborhood ties (T2 and T3) was inversely associated with the increase in the number of cafeterias in both models (e.g., −0.007 [−0.015, 0.000] for T2 and −0.008 [−0.015, 0.000] for T3 in Model 2). The other social capital indicators (i.e., norm of reciprocity, trust in the national government, and social participation) were unrelated to the change in the number of children’s cafeterias. The regression coefficients for all adjustment variables in the fully adjusted model (Model 2) are shown in Supplementary Table 7.

**Table 2:** Association between social capital and the number of children’s cafeterias.

		**Model 1**		**Model 2**	
	
		**b (95% CI)**	**β**	**b (95% CI)**	**β**
Trust in neighbors	T1	Ref.		Ref.	
	T2	0.006 (−0.001, 0.012)	0.088	0.006 (−0.002, 0.014)	0.099
	T3	0.008 (0.000, 0.016)	0.101	0.009 (−0.001, 0.019)	0.113
Norm of reciprocity	T1	Ref.		Ref.	
	T2	0.003 (−0.004, 0.009)	0.041	0.001 (−0.007, 0.009)	0.012
	T3	0.003 (−0.005, 0.010)	0.033	−0.003 (−0.013, 0.007)	−0.038
Trust in the national government	T1	Ref.		Ref.	
	T2	−0.002 (−0.009, 0.005)	−0.035	−0.002 (−0.010, 0.005)	−0.033
	T3	0.001 (−0.007, 0.009)	0.008	0.000 (−0.009, 0.009)	−0.001
Neighborhood ties	T1	Ref.		Ref.	
	T2	−0.008 (−0.015, 0.000)	−0.121	−0.007 (−0.015, 0.000)	−0.113
	T3	−0.008 (−0.016, −0.001)	−0.131	−0.008 (−0.015, 0.000)	−0.121
Social participation	T1	Ref.		Ref.	
	T2	−0.004 (−0.011, 0.003)	−0.062	−0.003 (−0.010, 0.005)	−0.039
	T3	−0.001 (−0.008, 0.006)	−0.013	−0.000 (−0.008, 0.007)	−0.006

CI: confidence interval.

b: non-standardized regression coefficient. β: standardized regression coefficient. T1: lowest tertile, T2: middle tertile, T3: highest tertile.

Adjusted for the population density of habitable land, the proportion of people aged 0–14 years, the proportion of people who graduated from university or graduate school, the proportion of single-parent households, the proportion of single-person households, the unemployment rate, the proportion of workers employed within their municipality of residence, the proportion of workers in the tertiary industry, social welfare expenditure, and the log-transformed number of children’s cafeterias per 1,000 individuals in 2020 included in both Models 1 and 2.

Model 1: Each social capital variable was separately added. Model 2: All five social capital variables were added.

As a sensitivity analysis, we restricted the sample to municipalities with ≥15 respondents (n = 356). The patterns of the associations were largely consistent with those of the main analysis (Supplementary Table 8).

## Discussion

This study investigated the association between social capital and the spread of children’s cafeterias using longitudinal data at the municipality-level. To our knowledge, this is the first longitudinal ecological study to examine the social/environmental factors contributing to the rise of community-based children’s cafeterias.

The substantial variability in changes across municipalities suggests that the diffusion of children’s cafeterias was not uniform. While some communities experienced rapid expansion, others showed stagnation or decline, pointing to disparities in local capacity to initiate or sustain such initiatives. The wide distribution of social capital indicators and structural characteristics further underscores the importance of contextual social environments in shaping community-based activities.

We observed that higher neighborhood trust was positively associated with an increase in the number of children’s cafeterias, and this positive direction remained after mutual adjustment for other dimensions of social capital. Social trust, a fundamental component of social capital [14], is crucial for fostering strong social relationships [15–17]. Therefore, for children’s cafeterias where diverse people gather, trust plays a pivotal role in their successful development and management. In other words, greater neighborhood trust in the community is crucial for fostering the social environment necessary for children’s cafeteria activities. Furthermore, cognitive social capital can enhance collective efficacy, enabling communities to address challenges effectively. When combined with high public awareness of the children’s cafeterias [1], collective efficacy may motivate community members to establish such facilities. The development and sustainability of children’s cafeterias often rely on support from community members, service users, and governments. Strong mutual cooperation can lead to better management and the long-term stability for these cafeterias.

A previous cross-sectional study from Japan found that stronger neighborhood ties were associated with a higher number of children’s cafeterias [10]. However, our longitudinal study showed the opposite: denser neighborhood ties were negatively associated with an increase in the number of cafeterias. In communities with dense neighbor connections, informal supports other than children’s cafeterias may be sufficient to prevent children from being alone or eating alone. For example, community members may be more likely to speak to neighborhood children if they are alone and children may have more opportunities to invite each other to their homes, reducing their chances of being alone. In contrast, in communities with weaker neighborhood ties, children’s cafeterias may serve as a place to supplement and foster connections in the community. Therefore, the increase in the number of cafeterias may have been greater in these communities.

This study had some limitations. First, we included only local municipalities with 10 or more respondents from the JACSIS study in our analysis to minimize high variability in the aggregated values. However, this might have introduced selection bias, as we analyzed only 512 out of 1,718 municipalities. We conducted a sensitivity analysis including local municipalities with five respondents or more respondents; however, the trend of the association remained similar. Second, social capital was measured in the JACSIS survey in 2020, during the coronavirus disease 2019 (COVID-19) pandemic, which considerably impacted daily activities and social relationships [18]. Notably, data collected during the COVID-19 pandemic may reflect anomalies not present in non-pandemic conditions. Therefore, the results should be interpreted with consideration of the potential aberrations introduced by the extraordinary circumstances of the pandemic period.

## Conclusion

This study suggests that cognitive and structural dimensions of social capital may be associated with the diffusion of children’s cafeterias across municipalities. In particular, cognitive and structural aspects of social capital appeared to operate in distinct ways. These findings underscore the importance of considering community social contexts when examining the spread of child-focused community initiatives.

## Supplementary information

**Additional file 1:** **Supplementary Table 1.** Characteristics of included and excluded municipalities. **Supplementary Table 2.** Characteristics of municipalities across tertiles of trust in neighbors. **Supplementary Table 3.** Characteristics of municipalities across tertiles of norm of reciprocity. **Supplementary Table 4.** Characteristics of municipalities across tertiles of trust in the national government. **Supplementary Table 5.** Characteristics of municipalities across tertiles of neighborhood ties. **Supplementary Table 6.** Characteristics of municipalities across tertiles of social participation. **Supplementary Table 7.** Full regression results for Model 2 examining the association between social capital and changes in the number of children’s cafeterias. **Supplementary Table 8.** Sensitivity analysis: Association between social capital and change in the number of children’s cafeterias in municipalities with ≥15 respondents (n = 356).

## Declarations

### Availability of data and material

The datasets used and analyzed in the current study are available from the corresponding author upon reasonable request.

## References

[1] Japan Kodomo Shokudo Support Center Musubie. Survey report on public awareness of children’s cafeteria in Japan 2023. 2023a. Available at: https://musubie.org/news/7686/. Accessed June 1, 2024.

[2] Japan Kodomo Shokudo Support Center Musubie. Survey report on number of children’s cafeteria in Japan 2023. 2023b. https://musubie.org/news/8560/. Accessed June 1, 2024.

[3] Shirasawa T, Ochiai H, Yoshimoto T, Matoba M, Sunaga Y, Hoshino H, Kokaze A. Effects of eating dinner alone on overweight in Japanese adolescents: A cross-sectional survey. BMC Pediatr. 2018;18(1):36. 29415682 10.1186/s12887-018-1041-y PMC5803896

[4] Kusano-Tsunoh A, Nakatsuka H, Satoh H, Shimizu H, Sato S, Ito I, Fukao A, Hisamichi S. Effects of family-togetherness on the food selection by primary and junior high school students: Family-togetherness means better food. Tohoku J Exp Med. 2001;194(2):121–7. 11642339 10.1620/tjem.194.121

[5] Machida D, Nagai Y, Yoshida T. Effects of kodomo shokudo (eateries for children) as examined by its staff: A qualitative study. Jpn J Health Educ Promot. 2018;26(3):231–7.

[6] Kurotani K, Shinsugi C, Chiba T, Yamaguchi M, Kachi Y, Takimoto H, Kondo N. An internet survey on “Children’s Cafeteria” for parents of elementary and junior high school students. Nihon Koshu Eisei Zasshi. 2019;66(9):593–602. 31588095 10.11236/jph.66.9_593

[7] Kawachi I, Berkman LF. Social capital, social cohesion, and health. In: Berkman LF, Kawachi I, Glymour MM, editors. Social Epidemiology (2nd ed.). New York: Oxford University Press; 2014. p. 290–319.

[8] Andrews R. Social capital and public service performance: A review of the evidence. Public Policy Adm. 2011;27(1):49–67.

[9] Altschuler A, Somkin CP, Adler NE. Local services and amenities, neighborhood social capital, and health. Soc Sci Med. 2004;59(6):1219–29. 15210093 10.1016/j.socscimed.2004.01.008

[10] Murayama H, Kurotani K, Tabuchi T. Social capital and the spread of children’s cafeterias (*kodomo shokudo*) in Japan: An ecological analysis. Asia Pac J Public Health. 2022;34(8):817–20. 35950628 10.1177/10105395221119416

[11] Ministry of Health, Labour, and Welfare. Comprehensive Survey of Living Conditions 2016. 2016. https://www.mhlw.go.jp/english/database/db-hss/cslc-report2016.html. Accessed June 1, 2024.

[12] Statistics Bureau. Statistical Observations of Municipalities 2020. 2020. https://www.stat.go.jp/english/data/s-sugata/index.html. Accessed June 1, 2024.

[13] Ministry of Internal Affairs and Communications. The 2020 population census. 2020. https://www.stat.go.jp/data/kokusei/2020/index.html. Accessed June 1, 2024.

[14] Putnam RD. Making Democracy Work: Civic Traditions in Modern Italy. NJ: Princeton University Press; 1993.

[15] Simpson JA. Psychological foundations of trust. Curr Dir Psychol Sci. 2007;16(5):264–8.

[16] Wieselquist J, Rusbult CE, Agnew CR, Foster CA. Commitment, pro-relationship behavior, and trust in close relationships. J Pers Soc Psychol. 1999;77(5):942–66. 10573874 10.1037//0022-3514.77.5.942

[17] Rempel JK, Holmes JG, Zanna MP. Trust in close relationships. J Pers Soc Psychol. 1985;49(1):95–112. 11474726

[18] Murayama H, Okubo R, Tabuchi T. Increase in social isolation during the COVID-19 pandemic and its association with mental health: Findings from the JACSIS 2020 study. Int J Environ Res Public Health. 2021;18(16):8238. 34443988 10.3390/ijerph18168238 PMC8394951

