# Supplementary material for: Social capital and the number of children’s cafeterias (kodomo shokudo) in Japan: a longitudinal ecological analysis
Source: Environ Health Prev Med. 2026 Jul 15;31:49. doi: 10.1265/ehpm.25-00366 (PMC13413657; doi:10.1265/ehpm.25-00366)
Supplement: Supplementary file 1 — Additional file 1: Supplementary Table 1. Characteristics of included and excluded municipalities. Supplementary Table 2. Characteristics of municipalities across tertiles of trust in neighbors. Supplementary Table 3. Characteristics of municipalities across tertiles of norm of reciprocity. Supplementary Table 4. Characteristics of municipalities across tertiles of trust in the national government. Supplementary Table 5. Characteristics of municipalities across tertiles of neighborhood ties. Supplementary Table 6. Characteristics of municipalities across tertiles of social participation. Supplementary Table 7. Full regression results for Model 2 examining the association between social capital and changes in the number of children’s cafeterias. Supplementary Table 8. Sensitivity analysis: Association between social capital and change in the number of children’s cafeterias in municipalities with ≥15 respondents (n = 356). [file ehpm-31-049-s001.docx]

| **Supplementary Table 1.** Characteristics of included and excluded municipalities | | | |
| --- | --- | --- | --- |
|  | Included municipalities  (n = 512) | Excluded municipalities  (n = 749) | p-value^a^ |
| Number of children's cafeterias per 1,000 individuals in 2020 | 0.04 ± 0.04 | 0.04 ± 0.07 | 0.794 |
| Number of children's cafeterias per 1,000 individuals in 2022 | 0.06 ± 0.05 | 0.07 ± 0.12 | 0.348 |
| Difference in the number of children's cafeterias per 1,000 individuals between 2020 and 2022 | 0.02 ± 0.03 | 0.03 ± 0.11 | 0.202 |
| Trust in neighbors (%) | 64.3 ± 19.3 | 66.3 ± 34.9 | 0.183 |
| Norm of reciprocity in the neighborhood (%) | 53.7 ± 19.8 | 57.0 ± 37.2 | 0.041 |
| Trust in the national government (%) | 41.3 ± 18.9 | 42.8 ± 37.5 | 0.336 |
| Neighborhood ties (%) | 11.1 ± 13.4 | 13.1 ± 26.9 | 0.090 |
| Social participation (%) | 21.7 ± 17.4 | 19.1 ± 30.1 | 0.057 |
| Population density of habitable land (individuals/km^2^) | 2966.6 ± 4094.0 | 1011.4 ± 1479.1 | <0.001 |
| % people aged 0–14 years | 12.1 ± 1.7 | 11.3 ± 2.4 | <0.001 |
| % people who graduated from university or graduate school | 18.3 ± 6.5 | 13.4 ± 5.3 | <0.001 |
| % single-parent households | 9.4 ± 1.3 | 9.9 ± 1.4 | <0.001 |
| % single-person households | 33.6 ± 7.4 | 30.2 ± 6.6 | <0.001 |
| Unemployment rate (%) | 3.8 ± 0.7 | 3.8 ± 1.1 | 0.388 |
| % workers in the tertiary industry | 67.7 ± 8.9 | 62.8 ± 8.9 | <0.001 |
| % workers employed within their municipality of residence | 57.6 ± 18.9 | 56.5 ± 19.6 | 0.300 |
| Social welfare expenditure (million yen) | 8132.3 ± 14524.4 | 1430.4 ± 1074.6 | <0.001 |
| Values represent mean ± standard deviation. | | | |
| a: t-test. | | | |

| **Supplementary Table 2.** Characteristics of municipalities across tertiles of trust in neighbors | | | |
| --- | --- | --- | --- |
|  | T1 | T2 | T3 |
| Population density of habitable land (individuals/km^2^) | 2827.3 ± 4221.3 | 3407.1 ± 4121.1 | 2078.0 ± 3726.9 |
| % people aged 0–14 years | 11.9 ± 1.6 | 12.4 ±1.8 | 11.7 ± 1.6 |
| % people who graduated from university or graduate school | 16.9 ± 5.8 | 19.8 ± 6.6 | 16.6 ± 6.5 |
| % single-parent households | 9.6 ± 1.2 | 9.2 ± 1.3 | 9.6 ± 1.3 |
| % single-person households | 33.3 ± 7.2 | 34.6 ± 7.6 | 31.7 ± 7.0 |
| Unemployment rate (%) | 3.9 ± 0.7 | 3.8 ± 0.6 | 3.8 ± 0.8 |
| % workers in the tertiary industry | 66.4 ± 8.3 | 69.8 ± 8.7 | 64.3 ± 8.6 |
| % workers employed within their municipality of residence | 56.0 ± 18.4 | 57.8 ± 19.6 | 59.5 ± 18.2 |
| Social welfare expenditure (million yen) | 6512.9 ± 15026.3 | 10531.5 ± 15971.7 | 4604.3 ± 7292.1 |
| Number of children's cafeterias per 1,000 individuals in 2020 | 0.04 ± 0.04 | 0.04 ± 0.04 | 0.05 ± 0.04 |
| Number of children's cafeterias per 1,000 individuals in 2022 | 0.06 ± 0.04 | 0.06 ± 0.05 | 0.07 ± 0.06 |
| Values represent mean ± standard deviation. | | | |
| T1: lowest tertile, T2: middle tertile, T3: highest tertile. | | | |

| **Supplementary Table 3.** Characteristics of municipalities across tertiles of norm of reciprocity | | | |
| --- | --- | --- | --- |
|  | T1 | T2 | T3 |
| Population density of habitable land (individuals/km^2^) | 2599.6 ± 3841.7 | 3477.3 ± 4457.2 | 2391.6 ± 3550.6 |
| % people aged 0–14 years | 12.1 ± 1.8 | 12.2 ± 1.7 | 12.0 ± 1.7 |
| % people who graduated from university or graduate school | 16.6 ± 5.5 | 19.7 ± 6.7 | 17.4 ± 6.7 |
| % single-parent households | 9.7 ± 1.2 | 9.2 ± 1.4 | 9.5 ± 1.3 |
| % single-person households | 32.9 ± 6.9 | 34.8 ± 7.8 | 32.2 ± 7.1 |
| Unemployment rate (%) | 3.9 ± 0.7 | 3.8 ± 0.6 | 3.8 ±0.7 |
| % workers in the tertiary industry | 66.3 ± 8.2 | 69.2 ± 8.8 | 65.9 ± 9.4 |
| % workers employed within their municipality of residence | 56.3 ± 19.2 | 58.5 ± 19.1 | 57.9 ± 18.1 |
| Social welfare expenditure (thousand yen) | 6388.1 ±15350.7 | 10649.0 ± 15991.2 | 5227.2 ± 7611.5 |
| Number of children's cafeterias per 1,000 individuals in 2020 | 0.05 ± 0.04 | 0.04 ± 0.04 | 0.04 ± 0.04 |
| Number of children's cafeterias per 1,000 individuals in 2022 | 0.07 ± 0.05 | 0.06 ± 0.04 | 0.06 ± 0.06 |
| Values represent mean ± standard deviation. | | | |
| T1: lowest tertile, T2: middle tertile, T3: highest tertile. | | | |

| **Supplementary Table 4.** Characteristics of municipalities across tertiles of trust in the national government | | | |
| --- | --- | --- | --- |
|  | T1 | T2 | T3 |
| Population density of habitable land (individuals/km^2^) | 2171.9 ± 3266.9 | 3530.5 ± 4618.1 | 2503.3 ± 3376.3 |
| % people aged 0–14 years | 12.0 ± 1.8 | 12.1 ± 1.6 | 12.2 ± 1.9 |
| % people who graduated from university or graduate school | 17.4 ± 6.4 | 19.1 ± 6.6 | 17.3 ± 6.2 |
| % single-parent households | 9.6 ± 1.3 | 9.3 ± 1.4 | 9.5 ± 1.2 |
| % single-person households | 32.2 ± 6.7 | 34.6 ± 8.0 | 32.8 ± 6.6 |
| Unemployment rate (%) | 3.8 ± 0.7 | 3.9 ± 0.7 | 3.8 ± 0.7 |
| % workers in the tertiary industry | 66.1 ± 9.0 | 68.9 ± 8.4 | 66.4 ± 9.4 |
| % workers employed within their municipality of residence | 55.8 ± 19.0 | 56.9 ± 19.3 | 61.0 ± 17.9 |
| Social welfare expenditure (thousand yen) | 4283.7 ± 7746.4 | 10494.4 ± 18437.2 | 6701.1 ± 7132.4 |
| Number of children's cafeterias per 1,000 individuals in 2020 | 0.04 ± 0.04 | 0.04 ± 0.04 | 0.04 ± 0.04 |
| Number of children's cafeterias per 1,000 individuals in 2022 | 0.06 ± 0.05 | 0.06 ± 0.05 | 0.06 ± 0.06 |
| Values represent mean ± standard deviation. | | | |
| T1: lowest tertile, T2: middle tertile, T3: highest tertile. | | | |

| **Supplementary Table 5.** Characteristics of municipalities across tertiles of neighborhood ties | | | |
| --- | --- | --- | --- |
|  | T1 | T2 | T3 |
| Population density of habitable land (individuals/km^2^) | 2590.0 ± 4219.9 | 4128.3 ± 4852.4 | 2110.0 ± 2820.8 |
| % people aged 0–14 years | 12.0 ± 2.0 | 12.1 ± 1.5 | 12.2 ± 1.7 |
| % people who graduated from university or graduate school | 17.4 ± 7.3 | 19.7 ± 6.9 | 17.5 ± 5.3 |
| % single-parent households | 9.6 ± 1.4 | 9.2 ± 1.4 | 9.5 ± 1.2 |
| % single-person households | 32.9 ± 8.0 | 35.5 ± 8.0 | 32.3 ± 6.1 |
| Unemployment rate (%) | 3.9 ± 0.7 | 3.9 ± 0.6 | 3.8 ± 0.7 |
| % workers in the tertiary industry | 66.6 ± 9.3 | 69.6 ± 9.1 | 66.5 ± 8.1 |
| % workers employed within their municipality of residence | 55.2 ± 17.8 | 55.4 ± 19.3 | 61.1 ± 18.8 |
| Social welfare expenditure (thousand yen) | 3274.7 ± 2345.0 | 12920.9 ± 21909.3 | 6521.6 ± 6685.2 |
| Number of children's cafeterias per 1,000 individuals in 2020 | 0.05 ± 0.05 | 0.04 ± 0.03 | 0.04 ± 0.04 |
| Number of children's cafeterias per 1,000 individuals in 2022 | 0.07 ± 0.06 | 0.06 ± 0.04 | 0.06 ± 0.04 |
| Values represent mean ± standard deviation. | | | |
| T1: lowest tertile, T2: middle tertile, T3: highest tertile. | | | |

| **Supplementary Table 6.** Characteristics of municipalities across tertiles of social participation | | | |
| --- | --- | --- | --- |
|  | T1 | T2 | T3 |
| Population density of habitable land (individuals/km^2^) | 1924.7 ± 2647.2 | 3600.0 ± 4697.0 | 3155.1 ± 4229.4 |
| % people aged 0–14 years | 12.2 ± 2.0 | 12.1 ± 1.5 | 12.1 ± 1.7 |
| % people who graduated from university or graduate school | 16.7 ± 6.3 | 19.5 ± 6.4 | 18.1 ± 6.5 |
| % single-parent households | 9.6 ± 1.4 | 9.3 ± 1.2 | 9.5 ± 1.4 |
| % single-person households | 32.6 ± 6.3 | 34.7 ± 7.7 | 33.2 ± 8.0 |
| Unemployment rate (%) | 3.9 ± 0.8 | 3.8 ± 0.6 | 3.9 ± 0.7 |
| % workers in the tertiary industry | 65.8 ± 8.2 | 69.3 ± 8.6 | 67.3 ± 9.4 |
| % workers employed within their municipality of residence | 60.0 ± 19.1 | 57.2 ± 19.0 | 55.7 ± 18.5 |
| Social welfare expenditure (thousand yen) | 4397.0 ± 4141.3 | 10294.6 ± 15065.6 | 8965.5 ± 19259.5 |
| Number of children's cafeterias per 1,000 individuals in 2020 | 0.04 ± 0.05 | 0.04 ± 0.03 | 0.04 ± 0.03 |
| Number of children's cafeterias per 1,000 individuals in 2022 | 0.07 ± 0.06 | 0.06 ± 0.05 | 0.06 ± 0.05 |
| Values represent mean ± standard deviation. | | | |
| T1: lowest tertile, T2: middle tertile, T3: highest tertile. | | | |

| **Supplementary Table 7.** Full regression results for Model 2 examining the association between social capital and changes in the number of children’s cafeterias | | |
| --- | --- | --- |
|  | β | p-value |
| Population density of habitable land | 0.039 | 0.690 |
| Proportion of people aged 0–14 years | 0.033 | 0.532 |
| Proportion of people who graduated from university or graduate school | -0.270 | 0.013 |
| Proportion of single-parent households | 0.106 | 0.236 |
| Proportion of single-person households | 0.028 | 0.782 |
| Unemployment rate | 0.048 | 0.346 |
| Proportion of workers in the tertiary industry | 0.151 | 0.056 |
| Proportion of workers employed within their municipality of residence | -0.083 | 0.307 |
| Social welfare expenditure | 0.048 | 0.346 |
| Log-transformed number of children's cafeterias per 1,000 individuals in 2020 in each local municipality | -0.111 | 0.018 |
| Trust in neighbors–T2 | 0.099 | 0.116 |
| Trust in neighbors–T3 | 0.113 | 0.072 |
| Norm of reciprocity–T2 | 0.012 | 0.847 |
| Norm of reciprocity –T3 | -0.038 | 0.556 |
| Trust in the national government–T2 | -0.033 | 0.585 |
| Trust in the national government–T3 | -0.001 | 0.986 |
| Neighborhood ties–T2 | -0.113 | 0.061 |
| Neighborhood ties–T3 | -0.121 | 0.044 |
| Social participation–T2 | -0.039 | 0.485 |
| Social participation–T3 | -0.006 | 0.913 |
| β: standardized regression coefficient. T1: lowest tertile, T2: middle tertile, T3: highest tertile. | | |

| **Supplementary Table 8.** Sensitivity analysis: Association between social capital and change in the number of children’s cafeterias in municipalities with ≥15 respondents (n = 356) | | | | | | |
| --- | --- | --- | --- | --- | --- | --- |
|  |  | Model 1 |  |  | Model 2 |  |
|  |  | b (95% CI) | β |  | b (95% CI) | β |
| Trust in neighbors | T1 | Ref. |  |  | Ref. |  |
|  | T2 | 0.003 (-0.005, 0.010) | 0.043 |  | 0.004 (-0.005, 0.013) | 0.071 |
|  | T3 | 0.008 (-0.001, 0.017) | 0.104 |  | 0.009 (-0.003, 0.020) | 0.113 |
| Norm of reciprocity | T1 | Ref. |  |  | Ref. |  |
|  | T2 | -0.001 (-0.009, 0.007) | -0.017 |  | -0.002 (-0.011, 0.007) | -0.031 |
|  | T3 | 0.000 (-0.010, 0.009) | -0.004 |  | -0.007 (-0.018, 0.005) | -0.087 |
| Trust in the national government | T1 | Ref. |  |  | Ref. |  |
|  | T2 | -0.004 (-0.012, 0.004) | -0.069 |  | -0.003 (-0.012, 0.006) | -0.052 |
|  | T3 | 0.001 (-0.008, 0.010) | 0.011 |  | 0.001 (-0.008, 0.011) | 0.019 |
| Neighborhood ties | T1 | Ref. |  |  | Ref. |  |
|  | T2 | -0.014 (-0.023, -0.004) | -0.230 |  | -0.012 (-0.022, -0.002) | -0.201 |
|  | T3 | -0.014 (-0.023, -0.004) | -0.232 |  | -0.012 (-0.022, -0.002) | -0.202 |
| Social participation | T1 | Ref. |  |  | Ref. |  |
|  | T2 | -0.006 (-0.013, 0.002) | -0.094 |  | -0.003 (-0.011, 0.005) | -0.056 |
|  | T3 | -0.004 (-0.013, 0.004) | -0.064 |  | -0.003 (-0.012, 0.006) | -0.048 |
| CI: confidence interval. | | | | | | |
| b: non-standardized regression coefficient. β: standardized regression coefficient. T1: lowest tertile, T2: middle tertile, T3: highest tertile. | | | | | | |
| Adjusted for the population density of habitable land, the proportion of people aged 0–14 years, the proportion of people who graduated from university or graduate school, the proportion of single-parent households, the proportion of single-person households, the unemployment rate, the proportion of workers employed within their municipality of residence, the proportion of workers in the tertiary industry, social welfare expenditure, and the log-transformed number of children's cafeterias per 1,000 individuals in 2020 included in both Models 1 and 2. | | | | | | |
| Model 1: Each social capital variable was separately added. Model 2: All five social capital variables were added. | | | | | | |
